# Chronic Cocaine Use and Parkinson’s Disease: An Interpretative Model

**DOI:** 10.3390/ijerph21081105

**Published:** 2024-08-21

**Authors:** Manuel Glauco Carbone, Icro Maremmani

**Affiliations:** 1 Division of Psychiatry, Department of Medicine and Surgery, University of Insubria, Viale Luigi Borri 57, 21100 Varese, Italy; manuelglaucocarbone@gmail.com; 2 VP Dole Research Group, G. De Lisio Institute of Behavioural Sciences, Via di Pratale 3, 56121 Pisa, Italy; 3 Saint Camillus International University of Health Sciences, Via di Sant’Alessandro 8, 00131 Rome, Italy; 4 Addiction Research Methods Institute, World Federation for the Treatment of Opioid Dependence, 225 Varick Street, Suite 402, New York, NY 10014, USA

**Keywords:** cocaine addiction, cocaine, Parkinsonism, Parkinson’s disease, extrapyramidal symptoms

## Abstract

Over the years, the growing “epidemic” spread of cocaine use represents a crucial public health and social problem worldwide. According to the 2023 World Drug Report, 0.4% of the world’s population aged 15 to 64 report using cocaine; this number corresponds to approximately 24.6 million cocaine users worldwide and approximately 1 million subjects with cocaine use disorder (CUD). While we specifically know the short-term side effects induced by cocaine, unfortunately, we currently do not have exhaustive information about the medium/long-term side effects of the substance on the body. The scientific literature progressively highlights that the chronic use of cocaine is related to an increase in cardio- and cerebrovascular risk and probably to a greater incidence of psychomotor symptoms and neurodegenerative processes. Several studies have highlighted an increased risk of antipsychotic-induced extrapyramidal symptoms (EPSs) in patients with psychotic spectrum disorders comorbid with psychostimulant abuse. EPSs include movement dysfunction such as dystonia, akathisia, tardive dyskinesia, and characteristic symptoms of Parkinsonism such as rigidity, bradykinesia, and tremor. In the present paper, we propose a model of interpretation of the neurobiological mechanisms underlying the hypothesized increased vulnerability in chronic cocaine abusers to neurodegenerative disorders with psychomotor symptoms. Specifically, we supposed that the chronic administration of cocaine produces significant neurobiological changes, causing a complex dysregulation of various neurotransmitter systems, mainly affecting subcortical structures and the dopaminergic pathways. We believe that a better understanding of these cellular and molecular mechanisms involved in cocaine-induced neuropsychotoxicity may have helpful clinical implications and provide targets for therapeutic intervention.

## 1. Introduction

Since the beginning of the current century, the spread of cocaine use has been an epidemic across the world. Cocaine is the second most consumed illicit drug in Europe, with prevalence and trends varying considerably between countries. According to the 2023 World Drug Report, 0.4% of the world’s population aged 15 to 64 report using cocaine; this number corresponds to approximately 24.6 million cocaine users worldwide and approximately 1 million subjects with cocaine use disorder (CUD) [1].

Cocaine is a psychostimulant that acts on the central nervous system (CNS), mainly through the involvement of the dopaminergic system. The mechanism of action of cocaine consists of blocking the dopamine transporter (DAT), resulting in an increase in the extracellular concentration of dopamine (DA) in the terminal areas of the dopaminergic system. The neurotransmitter is thus still able to stimulate the brain and prolong the sensation of pleasure users seek. Dopamine is one of the primary neurotransmitters involved in the pleasure and reward mechanisms in our brain [2].

The brain’s dopaminergic salience/reward pathways are crucial since they provide the pleasure drives to satisfy the physiological needs fundamental for the species’ survival through nutrition and reproduction by sharing the dimension of pleasure. They represent a “natural reward” and involve the release of DA in specific brain regions, such as the nucleus accumbens (NA) and frontal lobes, which play an essential role in the cognitive processes of aversion, motivation, reward, and self-reinforcement. However, the same release of DA and production of pleasure sensations can be triggered by “unnatural rewards” such as drugs (alcohol, cannabis, cocaine, heroin, etc.) and by compulsive behaviors such as pathological gambling, overeating, internet addiction, excessive sport, and high-risk activities [3].

Although it is well recognized that DA is the main neurotransmitter of the salience/reward pathways, other neurotransmitter pathways are crucially involved in reward and gratification processes. The “Brain Reward Cascade (BRC)” theory suggests that enkephalins, serotonin, norepinephrine, GABA, opioid, and cannabinoid neurons play a key role both directly and indirectly, potentially modifying dopamine metabolism, storage, release, and action [4].

Defects in various combinations of the genes for these neurotransmitters would seem to cause “dopamine resistance”, a form of sensory deprivation of the brain’s reward or pleasure mechanisms. This fact, in turn, results in a complex condition called Reward Deficiency Syndrome (RDS), and individuals with such characteristics are at risk for use to attain “unnatural rewards”.

This condition is characterized by a “hypodopaminergic trait”, which involves a deficit in the perception of rewards with the need to always seek new stimuli, out of the ordinary and with strong emotional content. The degree of this dysfunction can vary considerably, and the addiction could represent a manifestation of RDS [5].

The functional and neuroanatomical integrity of the brain structures involved, particularly the mesolimbic system with its amygdala, hippocampus, and NA components, guarantees an adequate perception of gratification, reinforcement, motivation, and happiness. Simplifying, BRC involves the release of serotonin, which stimulates enkephalins at the hypothalamic level. The inhibition produced by enkephalins on GABA at the level of the substantia nigra results in the release of DA in the nucleus accumbens. DA released into the synapse results in feelings of well-being and reduced stress [4,6].

The impairment of the gratification cascade can also be determined at the level of the receptor; genetic alterations involving the structure of the D_2_ receptor are supposed to be implicated in the onset of addictions [7].

### 1.1. Dopaminergic System

The dopaminergic system plays a central role in different cognitive and organic functions, such as the processing and execution of movement, motor control, spatial and recognition memory, maintenance of attention, memorization processes, motivation, reward, arousal, sleep regulation, feeding, olfaction, lactation, and reproductive behaviors [8,9].

The dopaminergic circuit participates significantly in the mechanisms of reward, learning, and acquisition of information coming from the environment with which the individual interacts. Dopaminergic transmission is fundamental in creating a state of motivation and gratification; it supports sensations of pleasure and reward, driving the individual to repeat a specific behavior. DA expresses a sense of “intentionality”; when its concentration in the synaptic cleft increases, the individual feels more oriented to “grasp” a potentially rewarding stimulus and becomes more focused on specific objectives.

At the same time, the brain structures interconnected to the dopaminergic system also process negative reinforcement, which is necessary for removing or avoiding unpleasant and uncomfortable stimuli or negative outcomes [10].

Three major dopaminergic pathways exist: the meso-cortico-limbic, the nigrostriatal, and the tuberoinfundibular pathways. Other dopaminergic pathways include the hypothalamo-spinal tract and the incerto-hypothalamic pathway [11].

The meso-cortico-limbic pathway starts from the ventral tegmental area (VTA) and projects on one side to the limbic structures (amygdala, NA, and hippocampus) and on the other to cortical areas (dorsolateral prefrontal cortex, DLPFC). In consideration of the selective afferents linked to the limbic system, the A10 area was designated for the cells of the VTA to differentiate them from neighboring cells [12]. The mesolimbic tract plays an important role in the genesis of emotional arousal and activation behavior in response to stimuli that predict a reward. This neuronal pathway acts as a sort of “reward switch”, signaling to other brain centers how pleasant an activity is [10,13]. The memorization of an action, therefore learning, is positively correlated with the extent of the emotional impact achieved (reward-related incentive learning) [14,15]. Specifically, in the incentive salience, which could be defined as the motivational value that is attributed to conditioned stimuli associated with rewards, and its aberrant attribution is a vulnerability factor for the development of psychopathologies related to addiction and impulse control disorders [16,17]. The mesocortical tract plays an important role in cognitive processes, emotional response, motivation, communication, and social interaction processes [18].

**Figure 1:**
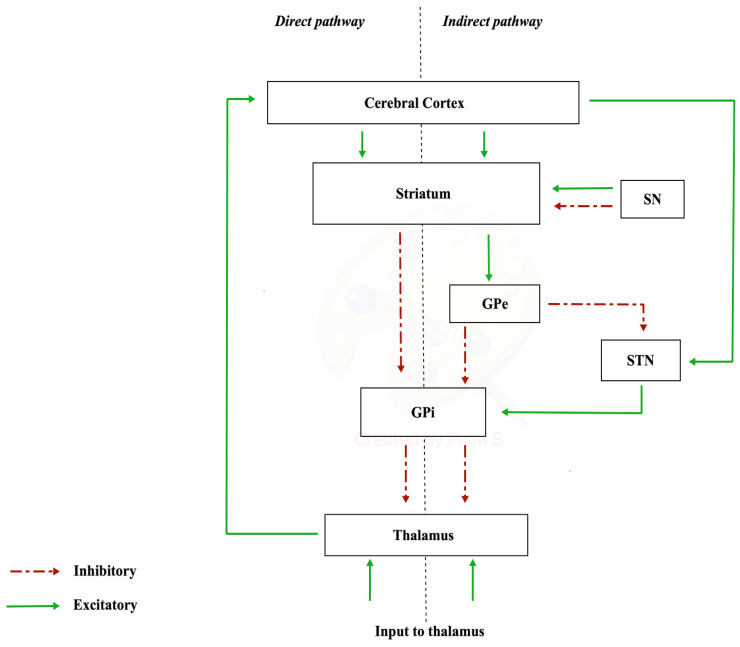
Direct and indirect pathways.

The nigrostriatal pathway originates from the neurons of substantia nigra (SN) (A9 cell group) and projects to the caudate nucleus and putamen (separated by the internal capsule), which together constitute the striatum. In the substantia nigra, there are different neuronal populations that give rise to two types of dopaminergic terminations: one which constitutes a dense and widespread innervation (matrix compartments) and another that forms specific densely innervated areas called striosome in the striatum [19]. The substantia nigra is made up of two portions, distinct according to a frontal plane: the pars compacta, characterized by a high density of dopaminergic neurons, located dorsally, and the pars reticulata, which houses GABAergic neurons, located ventrally [20]. The nigrostriatal dopaminergic pathway constitutes the so-called diffuse projection dopaminergic system, which is part of the extrapyramidal system responsible for the control of muscle tone and motor coordination [21]. The nigrostriatal pathway has a modulatory significance, as it has two types of dopamine-receptive neurons: D1-family receptor neurons, involved in the “direct pathway” of the basal ganglia, and D2-family receptor neurons, involved in the “indirect pathway”. In this way, the nigrostriatal pathway is able to excite the neurons of the direct pathway and inhibit those of the indirect pathway with a global significance of movement facilitation [22]. The diffuse projection dopaminergic system manages planning, initiation, and expression of movement planning, initiation, and expression of movement [23]. Communication via the “direct pathway” occurs with stimulation by the striatum. Excitation of the striatum has an inhibitory action on the internal or medial globus pallidus (GPi), resulting in the cessation of the inhibitory activity exerted by the GPi on the cells of the ventral lateral nucleus of the thalamus (VL). Disinhibition of the VL has the final effect of increasing the activity of the supplementary motor area (SMA). The “indirect pathway” involves other structures in the conduction of synaptic inputs, namely the external segment of the globus pallidus (GPe) and the subthalamic nucleus (STN) (Figure 1).

The degeneration of and consequent reduction in the number and activity of nigrostriatal dopaminergic neurons is the cause responsible for the symptomatic picture of Parkinson’s disease (PD).

The tuberoinfundibular pathway originates from the dopaminergic cell bodies of the arcuate and periarcuate nuclei of the hypothalamus (area A12) and projects to the pituitary gland (or hypophysis cerebri). The tuberohypophyseal system originates in the anterior part of the A12 area. It innervates the intermediate and posterior part of the pituitary gland, where it inhibits the secretion of melanocyte-stimulating hormone (α-MSH) and β-endorphin, the hormones oxytocin and vasopressin. DA released at this site inhibits prolactin secretion from anterior pituitary gland lactotrophs by binding to D_2_ receptors [24]. The neurons of the tuberoinfundibular system innervate the external layer of the median eminence where they are in close contact with the capillaries of the portal pituitary system.

The hypothalamo-spinal tract arises mainly from the paraventricular nucleus and lateral and posterior hypothalamic areas. This tract descends through the periaqueductal grey and adjacent reticular formation. The hypothalamo-spinal tract also connects the hypothalamus to the ciliospinal center of the intermediolateral cell column in the spinal cord (T1 to L2). It is also found in the pons, midbrain, lateral tegmentum of the medulla, and dorsolateral quadrant of the lateral funiculus [25].

The incerto-hypothalamic pathway is a short dopaminergic pathway from the zona incerta (ZI), located between the external medullary lamina and the subthalamic nucleus (STN), to the medial hypothalamus. It modulates fear and integrates autonomic and neuroendocrine responses to specific sensory stimuli, for example, during sexual behavior [26,27].

Furthermore, DA plays a key role in the Central Executive System (CES), a flexible system responsible for controlling and regulating cognitive processes [28]. It has the following functions: coordination of subordinate systems (slave systems); coordination of the execution of different tasks at the same time and recovery of strategies; selective attention and inhibition [29]. CES could be considered a coordinating system that regulates and guides behavior, monitors cognitive processes, and intervenes when they are not sufficient. Therefore, executive functions (EFs) are a set of cognitive processes that operate in a coordinated way to manage the information and actions necessary to achieve a goal. These high-level skills require the participation of different brain areas, in particular the prefrontal cortex, and are involved in decision making and adaptation activities [30,31].

Any dysfunction of the dopaminergic system consequently causes an altered functioning of the ES, affecting the EFs; this would imply an impairment of working memory, cognitive flexibility, fluid intelligence (e.g., reasoning and problem solving), attentional control, planning, prioritizing, scheduling, inhibitory control, and cognitive inhibition [32].

### 1.2. Cocaine Mechanism of Action

Cocaine is a substance that acts as a powerful central nervous system stimulant, vasoconstrictor, and anesthetic. It is an alkaloid obtained from the leaves of Erythroxylon coca, a plant native to South America, mainly Peru, Colombia, and Bolivia. Coca leaves have been used for centuries by the populations of South America as a tonic, against hunger, and as an anesthetic, especially during religious rituals that involved sacrificial practices by chewing the leaves or brewing teas.

The isolation of the active ingredient from cocaine leaves occurred in 1859, achieved by the German pharmacist Albert Niemann. It was considered safe at that time and was marketed on a large scale by the German pharmaceutical industry Merck as an antidepressant, in toothache drops, for the treatment of morphine and alcohol addiction, nausea pills, and the original “Coca-Cola” beverage [33,34]. Cocaine is a basic substance that can appear in different forms: (1) salified with hydrochloric acid (“hydrochloride salt”), where it takes the form of a powder; (2) free base, in solid form. Cocaine hydrochloride generally occurs as a fine white crystalline powder, is soluble in water, and is consumed mainly through the intranasal route (“sniffing”), orally, or intravenously.

“Freebase” is referred to by the common name “crack”; it is obtained through a chemical process that allows the transformation of the powder into a substance that can be smoked. The powder, or the hydrochloride salt of cocaine, is treated with ammonia or sodium bicarbonate and water; heating eliminates the hydrochloride, releasing the free base. It is typically consumed via inhalation; the solid mass is cracked into “rocks” that are smoked using glass or makeshift pipes [33,35].

Cocaine, like amphetamines and cathinones, is a psychostimulant. Its stimulating and euphoric effects are attributed to the fact that it is a non-selective inhibitor of the DAT, NET (norepinephrine transporter), and SERT (serotonin transporter), therefore blocking the reuptake of DA, norepinephrine, and serotonin, leading to an increase in the concentration of these neurotransmitters in various regions of the brain. Although the inhibition of the NET and SERT has been demonstrated, the magnitude of the binding and the factors influencing it are still poorly understood [36].

DAT collects the extracellular DA located in the synaptic cleft and transports it to the presynaptic neuron, where it is stored inside cellular vesicles.

**Figure 2:**
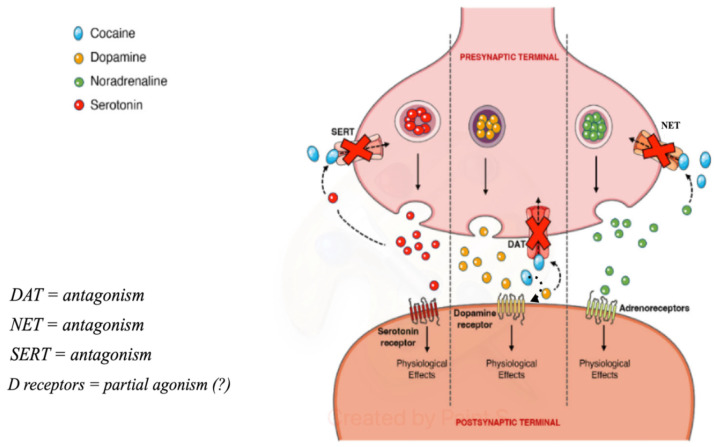
Pre-synaptic and post-synaptic effects of cocaine (from https://doi.org/10.3390/toxins14040278, (accessed on 3 May 2024) modified).

Cocaine binds tightly to DAT, preventing its regular functioning and causing an extracellular increase. Dopamine present in the synaptic cleft activates the post-synaptic dopamine receptors, activating dopaminergic transmission, which makes the drug rewarding and promotes the compulsive use of cocaine [2]. Furthermore, it has been demonstrated to function on post-synaptic dopamine receptors [37] (Figure 2).

Cocaine also appears to interact with serotonin receptors (5-HT receptors) [38]. In particular, the serotonin 5-HT_2C_ and 5-HT_2A_ receptors appear to regulate the strength of the behavioral response to cocaine. They could be mechanistically linked to cocaine-seeking [39]. The antagonism of cocaine on the 5-HT_2c_ receptors would contribute to some behavioral alterations such as motor hyperactivity and, in subjects with chronic use, would support the compulsive automatisms of seeking and taking the substance (the dimension of obsessive craving) [40]. Likewise, the stimulation of 5-HT_2A_ receptors could be involved in neurovegetative reactions following cocaine intake [41].

**Figure 3:**
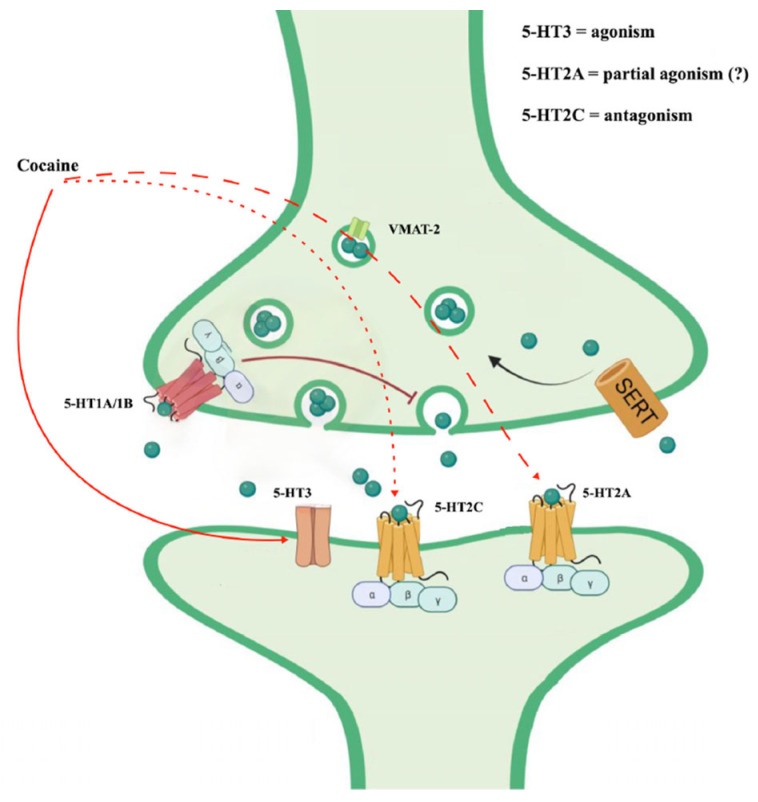
Cocaine effects on serotoninergic system (from https://doi.org/10.3390/ijms242216416, (3 May 2024) modified).

Furthermore, cocaine could act on salience through the activation of the opioidergic system mediated by the action on the 5-HT3 receptor. An antagonism on this receptor could reduce the automaticity of cocaine intake [42,43,44] (Figure 3).

Cocaine also binds sigma receptors, acting as a sigma ligand agonist. Sigma receptors are differentiated into two subtypes: Sigma-1 receptor (Sig-1R) and Sigma-2 receptor (Sig-2R). Although their distribution is predominantly in the CNS, they are also found at the peripheral level and differ from each other in their chemical and pharmacological profile, molecular weight, and transduction mechanism [45]. In particular, Sig-1R is an inter-organelle signaling molecule immersed in lipid rafts of the endoplasmic reticulum (ER), specifically the region of the ER that is strictly in contact with the mitochondria and to which it sends the activation signal through the release of calcium ions [46,47]. The Sig-1R receptor is attributed with activity in synaptic plasticity processes, a neuroprotective, neurotrophic, anti-amnestic, and anti-inflammatory action by reducing oxidative stress in the ER and mediating the activation of microglia and astrogliosis [48]. Recently, some studies have correlated alterations in the functionality of Sig-1R in the risk of onset of psychiatric and neurodegenerative pathologies as well as in addiction processes, such as cocaine misuse [49]. On the other hand, Sig-2 receptors, however, are less known, and their distribution is extended to various levels. In particular, they are overexpressed in various proliferating and quiescent tumor cells, both in the CNS and at the peripheral level (e.g., pancreas and breast); Sig-2R agonist ligands were found to induce tumor cell apoptosis via a caspase-dependent mechanism, resulting in increased intracellular Ca^2+^ [50].

Instead, cocaine’s effect on the glutamatergic site has long been demonstrated. In fact, cocaine induces alterations in the protein expression of NMDA receptor subunits, structural changes in their composition and synaptic distribution, and, finally, altered coupling of NMDA receptors to D_1_ dopamine receptor-mediated signals.

The effects of cocaine appear to be strongly related to the drug addiction history of the subject, the methods of intake, the duration of use of the substance, and any time intervals of abstinence [51].

Different studies have highlighted how the rewarding effect of cocaine and possibly craving during withdrawal followed by relapse may be related to a region-specific activation of the µ-opioid receptor system. At the same time, the rewarding effect of cocaine could be reduced through a region-specific activation of the κ-opioid receptor/dynorphin system, which would increase its stress-inducing properties and facilitate the risk of relapse [52,53].

Cocaine, acting as an antagonist, blocks the rapid entry of sodium into nerve cells, and its anesthetic properties are due to this. On cardiac cells, it blocks the rapid entry of sodium and the slow entry of calcium into the cells and blocks the return of potassium into the cells undergoing repolarization. Depending on the effects of cocaine at the CNS and peripheral levels, to which is added increased platelet aggregation, its adverse acute and chronic cerebrovascular consequences are well understood (tachycardia, hypertension, vasoconstriction, supraventricular arrhythmias, possible cardiomyopathy, increased risk of stroke) [54,55].

### 1.3. Cocaine Use Disorder (CUD)

Cocaine use remains a significant health problem with severe socio-economic consequences worldwide [56]. According to the most recent World Drug Report, 22 million individuals aged 15–64 reported cocaine use in the past year, of whom 73% were men. In Latin America, people entering drug treatment report cocaine as the most frequent primary drug of use. In the European Union, it represents the second most used substance, only after cannabis [1]. In the United States, about 1.5 million individuals meet the Diagnostic and Statistical Manual for Mental Disorders (DSM-5) criteria for cocaine use disorder (CUD) [1,57,58].

CUD, like all substance use disorders, tends to be chronic and relapsing in nature [59]. Epidemiological studies show high rates of comorbidity with mood disorders, anxiety, and personality disorders in cocaine users and an increase in the consumption of this substance in patients who previously had primary psychotic and anxiety disorders. Studies involving samples of cocaine-dependent subjects indicate comorbidity rates for mood disorders between 30 and 50%. Bipolar disorder is a morbid condition frequently associated with subjects with CUD (20–30%). The association between cocaine use, panic attacks, generalized anxiety, post-traumatic stress disorder (PTSD), and attention deficit hyperactivity disorder (ADHD) is also important but, in many cases, difficult to measure in certain percentages, especially due to multiple comorbidities. As regards social phobia, however, the lifetime prevalence in this population is around 10–15% [60,61,62,63,64,65,66,67].

As we have already said above, chronic use of cocaine favors the onset of neuroinflammatory processes due to increased oxidative stress related to alterations in the functionality of the mitochondria, the ER, and altered activation of microglia and astrogliosis processes, which cause excitotoxicity phenomena and neurotoxicity [68,69]. This condition of dysregulated cerebral homeostasis could explain, even if only in part, the proposed increased risk of incidence of neurodegenerative diseases in cocaine users, since these neurotoxic processes have also been associated with the genesis and/or progression of many neurodegenerative brain disorders, such as PD and Alzheimer’s disease (AD) [70].

### 1.4. Parkinson’s Disease (PD)

Parkinson’s disease is an idiopathic degenerative disease of the nervous system with a slow but progressive evolution. It mainly affects some functions, such as movement control and balance, but it can also present or onset with non-motor system manifestations or cognitive alterations. PD is the most frequent movement disorder and the second most common neurodegenerative disorder after AD. It is found in both sexes, with a slight prevalence in males. The average age of onset is around 58–60 years, but approximately 5% of patients can present a young onset between 21 and 40 years. Before the age of 20, it is extremely rare. Over 60, it affects 1–2% of the population, while the percentage rises to 3–5% when the age is over 85 [71,72].

PD has an anatomopathological substrate of degeneration of the dopaminergic neurons of the substantia nigra pars compacta (SNpc), located in the midbrain, with a gradual progression and a prolonged course. A histological hallmark of PD is represented by the presence of Lewy bodies (named after the neurologist F.H. Lewy, who identified them in 1912), which accumulate in particular in the SNpc. Lewy bodies are spherical inclusions, detectable on histological examination, mainly formed by aggregates of α-synuclein, an insoluble protein.

However, PD is characterized by more widespread pathology in other brain regions and involves non-dopaminergic neurons. The diagnosis of Parkinson’s disease is based not only on the clinical examination but also on the patient’s clinical and family history and on the evaluation of neurological symptoms and signs.

The clinical suspicion of PD is based primarily on the onset of motor features, such as muscular stiffness characterized by resistance to passive movements, resting tremor (often asymmetric) that can increase in an anxiety state, and bradykinesia, which causes difficulty starting and finishing movements. These symptoms then result in balance disorders, awkward gait, and curved posture.

Non-motor features, which include olfactory dysfunction, sleep disturbances (often REM sleep behavior disorder, insomnia, restless legs), mood changes, anxiety, panic attacks, apathy, fatigue, impaired color discrimination, and constipation, can develop years before motor deficits (Premotor Stage). On the other hand, during later stages of the disease, important autonomic dysfunction (blood pressure alterations, thermoregulation, sweating, urological and gastrointestinal disorders), somatosensitive alterations (hyperalgesia and dysesthesia), behavioral alterations, and cognitive decline can appear [73].

PD is the result of the interaction between numerous environmental factors to which the patient is exposed during his life (toxic substances, drugs, lifestyle, infections) and an inherited genetic predisposition. The presence of a genetic predisposition is confirmed by the fact that 10% to 16% of patients with PD report at least one affected first-degree relative (children, parents, brothers, sisters). Numerous studies on genetic aspects are beginning to identify the various genes involved in predisposition to PD. The genes identified in these families so far total eleven (called PARK), of which six have actually been identified: alpha-synuclein (PARK-1), parkin (PARK-2), ubiquitin hydrolase (PARK-5), PINK1 (PARK-6), DJ-1 (PARK-7), LRRK2 (PARK-8) [74].

The Parkin, PINK-1, and DJ-1 genes function in a recessive mechanism and typically cause juvenile-onset PD before age 40. In these cases, the probability of developing the disease is high even if the age of onset cannot be known with certainty. The genes that function by a dominant mechanism are α-synuclein and LRRK2. Both appear to be involved in typical late-onset forms of PD.

Among the pathogenetic mechanisms responsible for the neurodegenerative process, mitochondrial dysfunction seems to play a leading role and has been found in cases of both familial and sporadic PDs. Given the importance of the numerous functions carried out by mitochondria and the frequency with which these organelles are damaged, as in the case of chronic exposure to cocaine, their correct turnover is critical for the survival of the cell. The elimination of dysfunctional mitochondria occurs through a form of selective autophagy called mitophagy. The main mitophagy pathway is mediated by the coordinated action of PINK1 and Parkin proteins, which are mutated in some forms of juvenile-onset autosomal recessive PD. Only recently have the neuroprotective action of astrocytes and their potential involvement in the pathogenesis and progression of PD been taken into consideration. The action of PINK1 and Parkin appears to be more extensively regulated in astrocytes than in neurons, suggesting a particular relevance of mitophagy in this type of glial cell. The fragmentation of damaged and incorrectly degraded mitochondria triggers the pro-inflammatory activation of astrocytes and microglia, a phenomenon that aggravates neurodegeneration in pathological conditions. Among the numerous functions of astrocytes, their ability to degrade damaged axonal mitochondria coming from adjacent neurons has also emerged through a process called transcellular mitophagy (or trans-mitophagy). Trans-mitophagy has been detected in different cellular contexts and recently observed in striatal astrocytes in the presence of degenerating dopaminergic neurons [75,76,77,78,79]. This scientific evidence has led many authors to consider PD as a primary disease of the mitochondrion [80].

Some researchers have suggested defects in protein clearance pathways that cause alpha-synuclein protein aggregation and promote the formation of Lewy bodies. Since this pathology is characterized by the presence of this “misfolded protein” or incorrect folding that leads to its accumulation, PD is included in the group of proteinopathies [81]. The ubiquitin–proteasome system (UPS) is the main pathway responsible for the degradation of cytosolic, nuclear, and ER proteins via a small protein called ubiquitin. The malfunction of the UPS causes serious cellular alterations and, if it persists, often leads to cell death. In PD, as in other neurodegenerative processes, we very frequently find an accumulation of ubiquitinated proteins [82].

Other researchers have developed the “dual hit” theory to explain the pathophysiological progression of PD. This hypothesis suggests that a pathogenic agent, probably viral, enters the brain through two routes: the nasal one, with anterograde progression in the temporal lobe, and the gastric one, secondary to the swallowing of nasal secretions in saliva. Pathological support for this hypothesis comes from the identification of Lewy bodies in the olfactory bulb and enteric plexus of the stomach. Notably, PD patients often have prodromal olfactory deficits [83,84,85].

## 2. An Interpretative Model of the Relationship between CUD and PD

The use of cocaine represents a worldwide problem of burning and current social relevance, a source of very serious concern and a negative impact on citizens, institutions, and, more generally, the entire community [86]. From the available data cited in the paragraph above, cocaine use in Europe, although affecting only a relatively small part of the general population, is constantly increasing every year Drugs, 2006 [87]. Even considering all their limitations, the epidemiological data unequivocally indicate how widespread cocaine is now in all social environments; how its use is massive and has taken on very diverse forms; how it causes accidents of all kinds and serious and increasingly evident damage to the people who consume it and to the community; how the trafficking connected to it is decisive for the widespread diffusion and strengthening of large criminal holding companies operating on a global scale, with significant repercussions at an institutional, economic–financial, and social level [1].

It must also be considered that cocaine interacts with any basic condition (medical and psychopathological), aggravating it to extreme consequences in terms of severity and recovery. Thus, dysfunctional temperamental and personological traits are made dramatically explosive, and variants of depressive syndromes, bipolar spectrum, and psychotic spectrum associated with substance use are much more difficult to recognize and manage. At the same time, it can be hypothesized that chronic cocaine consumption, in relation to the proven negative organic effects (on the CNS and on several systems of the human body), may cause, favor, or anticipate the onset of neurodegenerative pathologies, perhaps with different clinical phenotypes. It is fair to believe, therefore, that it is not at all an overstatement to say that the growing widespread diffusion of cocaine and its chronic use can significantly change the modalities of clinical presentation, the course, the evolutionary trajectory, the treatment (the prognosis, the costs, the outcomes) of neuropsychiatric syndromes.

Below, an interpretative model on the possible relationship between CUD and increased risk of PD will be explained, with the identified mechanisms listed in subparagraphs. This interpretative model has the task of highlighting how cocaine can act pathogenetically on different levels and induce brain damage, to a greater or lesser degree, favoring the onset of movement disorders and/or psychiatric syndromes.

We believe that it is fundamental in the clinical psychiatric field and in addiction medicine to take these aspects into consideration and, above all, the related therapeutic implications.

### 2.1. Dopaminergic Neurotransmission Alterations

As previously mentioned, the administration of cocaine causes widespread alterations in the dopaminergic system. These effects are more evident in the basal nuclei (striatum, above all), the limbic system, and the prefrontal cortex. In acute form, cocaine blocks DAT, located in the pre-synaptic nerve terminal, leading to a high extracellular increase in DA [52].

DAT is the protein that gathers DA from the synaptic cleft in the pre-synaptic neuron and transports it into the cytoplasm. DAT activity is dynamically regulated by endocytic trafficking and, depending on the signals emitted by the cell, endocytic (speed and frequency of DA entry) and post-endocytic processes are established. The latter involves storage processes within granular vesicles, mediated by vesicular monoamine transporter 2 (VMAT-2) activity, and degradation carried out by monoamine oxidase (MAO) or catechol-O-methyl transferase [88,89].

Contextually, cocaine could act as a partial agonist on post-synaptic dopamine receptors, supporting downstream dopaminergic transmission [90]. At the same time, cocaine, by binding in part to SERT and NET, increases extracellular levels of serotonin and norepinephrine [36]. The widespread high increase in all monoamines, predominantly DA, would seem to be responsible for the euphoric, disinhibiting, impulsive effects of cocaine as well as its neurovegetative, metabolic, and motoric side effects [34].

In chronic use, cocaine appears to cause a series of neuroadaptive changes involving cellular protein expression at pre-synaptic and post-synaptic levels. In vitro, post-mortem studies carried out in the brains of subjects affected by CUD [91,92,93] and in vivo in acutely abstinent cocaine addicts have allowed the identification of DAT upregulation phenomena [94].

Similarly, chronic cocaine use is associated with increased neuronal synthesis of synucleins, a family of soluble pre-synaptic proteins. They exert a significant regulatory effect on the synaptic vesicle transport processes [95,96,97,98]. Specifically, α-synuclein directly binds and functionally couples DAT, increasing DA uptake and accelerating DA-induced apoptosis [99]. The α-synuclein mRNA of cocaine users was increased in the VTA and SN compared to a control group of age-matched subjects who did not use the drug [100].

This neuroadaptive modification has functional relevance for regulating intracellular DA trafficking, which varies following cocaine intake. The discovery of these changes in neuronal protein expression in the VTA and SN would seem to suggest that these structures are particularly “affected” by cocaine and are fundamental induction sites for cocaine-induced increases in α-synuclein. On the other hand, excessive cytoplasmic formation of α-synuclein induces dose-dependent cytotoxic effects that can lead to neurodegeneration phenomena involving dopaminergic neurons and other neuronal populations [101].

In midbrain DA neurons, α-synuclein is overexpressed by cocaine users, and that may put cocaine addicts at risk for degenerative changes in DA neurons, including motor abnormalities of the PD [100].

It is also possible to hypothesize that similar phenomena may occur in NETs and SERTs. This would imply an overall reduction in catecholamine reuptake and their progressive reduction in storage, synthesis, and release.

In response to the elevated DA levels in the synaptic cleft, chronic users might downregulate post-synaptic dopamine receptors as a compensatory mechanism [102]. This adaptive phenomenon could explain the detected hypertrophy of the putamen, which, to compensate for the reduced efficiency of post-synaptic dopamine receptors, would increase its activity to maintain adequate dopaminergic transmission [103].

Chronic cocaine use seems to lead, over time, to an overstimulation of monoaminergic circuits, especially dopaminergic ones, and a reduced capacity for degradation and metabolization of these neurotransmitters. The “stress” to which the dopaminergic pathways are subjected would gradually and progressively lead to a loss of efficiency in neurotransmission; the speed and extent of dysregulation of the dopaminergic system would depend both on the modalities and quantities of substance abuse and on the physiological characteristics of the individual’s homeostasis systems [104].

In this sense, an inverted-U-shaped relationship between dopamine levels and performance in subjects with PD has been identified. The regional striatal topography of nigrostriatal denervation and the individual genotype are critical factors determining the relative baseline position on this inverted U curve. It follows that the prolonged use of cocaine, but more generally of dopaminergic pharmacotherapies, in relation to the presence of individual gene polymorphisms, can affect the dopaminergic pathways (mesocortical, mesolimbic, nigrostriatal, tuberoinfundibular, etc.) in a comparable, inverted-U dose–response relationship (“Dopamine Overdose hypothesis”) [104].

Taken together, these combined effects (pre- and post-synaptic) progressively attenuate the acute increase in DA and in the dopaminergic transmission associated with cocaine intake. In the long term, there is a deficiency of DA in the striatum, VTA, and prefrontal cortex, as in order to compensate for this condition, they attempt to increase the reuptake and synthesis of DA until progressively reducing the neuronal response to DA and the dopaminergic transmission [105]. In support of this hypothesis, several studies conducted in vivo and post-mortem in subjects with cocaine use disorder show a reduction in dopaminergic neuronal components (striatal and mesencephalic), suggesting a loss in the number of brain dopaminergic cells [106].

In support of the hypothesized alteration of the integrity of the dopaminergic pathways in subjects with cocaine use, Parkinsonian-type motor control anomalies of the subclinical entity were detected in subjects with previous cocaine abuse, abstinent for a period of at least three months [107]. Similarly, neuroimaging studies have highlighted brain morpho-structural and functional changes, possibly permanent, in subjects with a previous cocaine use disorder who have been abstinent for at least 4–9 months [108,109,110].

### 2.2. Mitochondrial Dysfunction and Neurotoxicity

As previously mentioned, the chronic use of cocaine, and more generally of psychostimulants, is related to altered mitochondrial function and an increase in the formation of reactive oxygen species (ROS) such as hydroxyl and hydrogen peroxide. The increase in oxidative stress and the reduced mitochondrial anti-aging and antioxidant ability lead to an increase in DNA oxidation and lipid peroxidation processes within the target pathways. This results in an increased state of neurotoxicity, especially in the structures most “affected” by cocaine and more sensitive to oxidative stress, such as the striatum and the NAc, with relative damage to the striatal nerve terminal [111]. At the same time, it is now well known that the pathogenesis of PD, both familial and sporadic, is closely connected to mitochondrial dysfunction and increased oxidative stress [80].

Specifically, various port-mortem studies conducted on subjects suffering from PD have shown a dysfunction of the activity of the mitochondrial complex I. A lower efficiency of the activity of this complex would increase the production of superoxide radicals with a relative increase in oxidative stress. This condition, together with reduced functionality of the enzymatic and non-enzymatic anti-oxidative defense system (superoxide dismutase, catalase, glutathione, etc.), would favor processes of neurotoxicity, cellular apoptosis, and consequent onset or progression of neurodegenerative pathologies [112].

Furthermore, an increased metabolism and intracellular turnover of DA leads to a greater production of superoxide radicals and hydrogen peroxides with elevation of the state of oxidative stress in the neurons of the dopaminergic pathways [113].

In vivo and in vitro models of PD were reproduced through the administration of the dopaminergic neurotoxin 6-hydroxydopamine (6-OHDA). This molecule induces processes of dopaminergic neurotoxicity through the formation of highly reactive dopamine quinone and ROS [114].

The elevation of oxidative stress, due to the ROS-mediated oxidation of methionine residues, facilitates the aggregation of α-synuclein into amyloid fibrils, reduces proteasomal degradation, and promotes the formation of Lewy bodies, the main histopathological hallmark of PD, responsible for various neurotoxic effects on neurons of the dopaminergic pathways.

Alpha-synuclein aggregates influence the activity of VMAT-2, interfering with its functioning. As previously mentioned, VMAT-2 is located in the cytoplasm of the pre-synaptic neuron and has the task of transporting monoamines (DA, 5-HT, NE, and histamine) from the cytoplasm into the granular vesicles [115,116].

V-MAT-2 exerts a protective role, as by removing free dopamine at the cytoplasmic level, it reduces the formation of ROS [117,118,119]. In line with this consideration, in the MPTP non-human primate model of PD, a potential reduction in VMAT-2 functionality and the related dysfunctional dopamine storage capacity represents a key pathogenic event in the retrograde degeneration of the dopaminergic nigrostriatal pathway. For this reason, the aggregation of α-synuclein amyloid fibrils at VMAT-2, induced by high levels of oxidative stress, would culminate in a series of events resulting in the neurodegeneration of dopaminergic neurons [120].

The VMAT-2 reduction in the striatum is the first pre-synaptic DA terminal marker prior to terminal degeneration; impaired DA storage capacity is sufficient to cause DA-mediated oxidative stress and nigrostriatal DA system degeneration [120]. Logically, environmental factors and/or genetic mutations that reduce the VMAT-2 system functionality may increase susceptibility to PD.

Moreover, the formation of α-synuclein aggregates interferes with the function of proteasomes and lysosomes, which in turn further promotes the aggregation of α-synuclein oligomers [121]. As discussed previously, some sporadic autosomal recessive forms of PD are related to functional alterations of the PINK1/Parkin system; PINK1 and Parkin are two proteins involved in regulating the mitophagy process.

PINK1 is a serine/threonine kinase, while Parkin is an E3-ubiquitin ligase that intervenes in the ubiquitination of proteins destined to be degraded. Parkin is normally a cytosolic protein and is recruited to non-functioning mitochondria in a PINK1-dependent manner.

Damaged mitochondria present a drastic reduction in mitochondrial membrane potential, resulting in the accumulation of PINK1 on the mitochondrial outer membrane. PINK1 phosphorylates ubiquitin present on the mitochondrial surface, resulting in recruitment and activation of the E3 ligase enzyme Parkin. The PINK/Parkin bond constitutes a “tag” that signals that the mitochondrion must be destroyed.

Loss of Parkin function makes neurons more vulnerable to cytotoxic insults, such as those mediated by the accumulation of α-synuclein. Parkin not only removes α-synuclein, but by constituting the Parkin/α-synuclein complex, it plays an active role in activating mitophagy [122,123]. The impairment of the Parkin/PINK1 system implies a mitochondrial dysfunction contributing to the increase in oxidative stress and proteasomal/lysosomal dysfunction, characteristic of neurodegenerative processes such as PD [124,125]. Furthermore, the evolution and pathogenic course of PD appears to be supported by chronic neuroinflammation processes [126]. Specifically, in the SN of PD subjects, abundant reactive microglia, and CD4+ and CD8+ T cells were detected.

The activation of lymphocytes and microglia leads to an inflammatory state due to the release of pro-inflammatory cytokines such as interleukin (IL)-1β, IL-6, tumor necrosis factor (TNF)-α, perforins, granzymes, nuclear factor κB (NFκB), and inducible nitric oxide synthase (iNOS) [127,128]. This inflammatory state would further fuel the state of oxidative stress of the neurons of the dopaminergic pathways through the formation of superoxide radicals via the NADPH oxidases [70,129,130,131].

Interestingly, Vicente-Rodriguez et al. identified 23 proteins differentially regulated in the mouse striatum by chronic cocaine exposure depending on the endogenous expression of pleiotrophin (PTN) [132]. PTN is a small cationic protein with potent mitogenic and angiogenic activity; it has been associated with a wide range of critical biological events, including neurite growth stimulating activity, neuron development, oligodendrocyte differentiation, bone development, inflammation, cancer metastasis, and tissue repair [133]. The proteins identified in the study are related to neurodegeneration processes, drug-induced neurotoxicity, and oxidative stress. Acute and chronic treatments with cocaine by differentially influencing the activity of these proteins could favor the onset of neurodegenerative processes. The detrimental effects of cocaine on the protective role of PTN are indeed more evident in PTN−/− mice, highlighting its function in preventing cocaine-induced neural alterations [132].

Finally, the chronic use of cocaine, by interfering with the activity of the SIg-1 receptor, triggers inflammatory processes, supported by the activation of microglia, causing an alteration in the functionality of the blood–brain barrier (BBB), with an increase in the risk of neurotoxicity [134].

### 2.3. Morphological Plasticity Alterations and Dysregulation of Neurogenesis

Chronic cocaine exposure results in an increase in spine density (spinogenesis) in medium spiny neurons (MSNs) [135,136,137,138]. These morphological changes influence mesolimbic, mesocortical, and nigrostriatal pathways. They may contribute to the development of compulsive patterns of drug-seeking behaviors, reduced behavioral sensitization (a neuroadaptive process characterized by an increase in a particular behavior after repeated exposure to drugs or other stimuli), and increased risk of psychomotor symptoms [139,140]. The D1-positive “direct pathway” neurons would seem to be more sensitive to cocaine-induced changes in spine density than D2-containing “indirect pathway” neurons [141,142]. Spine density changes could be related to calcium-mediated regulation of myocyte enhancer factor 2 (MEF-2). MEF-2 proteins regulate excitatory synapses by promoting activity-dependent synaptic pruning [143,144]. Chronic cocaine exposure causes an abnormal increase in DA with an overactivation of the D1 receptor and its cAMP-dependent signaling cascade. Furthermore, the activity of the transcription factor DeltaFosB leads to increased synthesis of cyclin-dependent kinase 5 (Cdk5), which reduces MEF-2 activity and results in long-term adaptive changes in spine density in NAc neurons expressing the D_1_ receptor [139,140].

Finally, as mentioned in the previous paragraphs, the use of cocaine causes various acute and chronic effects on the autonomic, cardiac, endothelial, and coagulation systems [145]. In particular, the use of cocaine is associated with an increase in blood pressure and heart rate, a slowdown in the propagation of the electrical stimulus in the myocardium, coronary vasoconstriction, vasospasm of the microcirculatory vessels, platelet hyperactivation with increased aggregation, etc. Furthermore, the use of cocaine, by triggering a greater condition of oxidative stress and through the activation of the caspase pathways, would lead the cerebral vascular smooth muscle cells to rapidly activate apoptosis processes, favoring cerebral ischemic and hemorrhagic processes. The induction of apoptosis in the smooth muscle cells of the cerebral vessels appears to have a dose-dependent correlation with cocaine.

These widespread systemic effects exponentially increase the risk of cerebral microvascular damage, cerebral vascular toxicity, and cardio- and cerebral-vascular events (ischemic and hemorrhagic), with a consequent increase in the risk of lesions affecting the central nervous system [146].

Cocaine exerts a cumulative pathogenic effect on brain tissue. Indeed, the duration of cocaine use correlates inversely with the grey matter volume of prefrontal areas [147]. Cocaine-addicted patients had a decreased grey matter density in frontal and temporal areas, orbitofrontal cortex, limbic cortex, anterior cingulate, insular, and superior temporal cortex, compared to non-cocaine users [148]. Cocaine-addicted patients also showed frontal white matter abnormalities and reduced cerebellum volume [149,150,151]. An animal study has also reported cocaine-induced microscopic lesions in the cerebellum [152].

Neuroimaging studies have highlighted morpho-structural alterations in subjects with chronic cocaine use, specifically cortical atrophy of the temporal lobe, a reduction in the volume of grey matter in the cortical and subcortical regions, and a release of the connectivity of the frontal white matter. These alterations would appear to be directly correlated with the duration of continuous use of the substance and with the age of the subject, indicating an effective widespread cerebral neurodegenerative action of cocaine [153] [154]. Furthermore, in cocaine addicts, there are impairments in coordination, balance, and the execution of fine and precise movements, suggesting a potential cerebellar dysfunction [155,156,157].

## 3. Discussion

**Figure 4:**
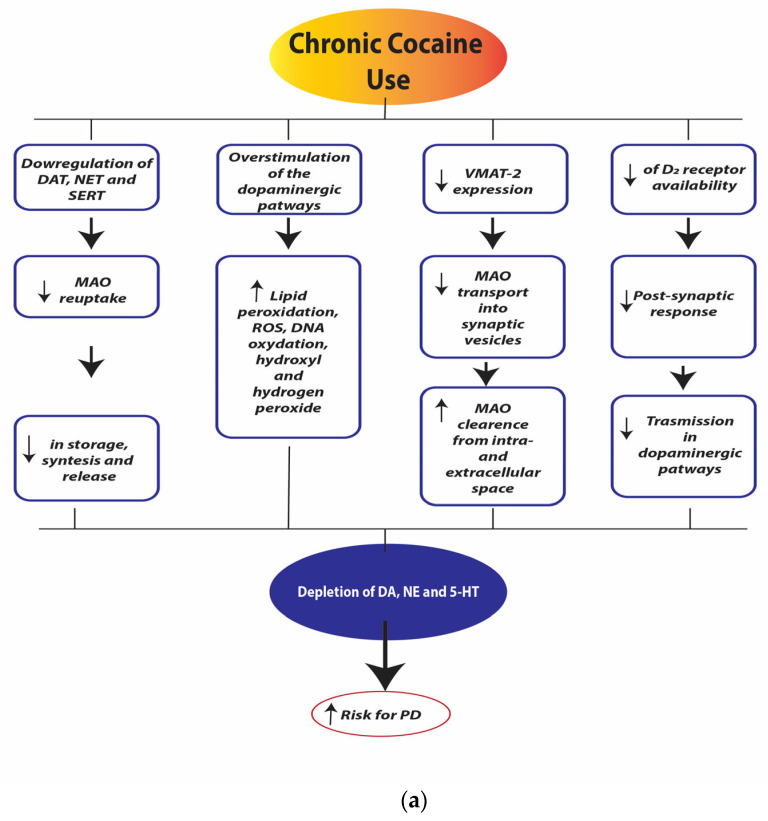

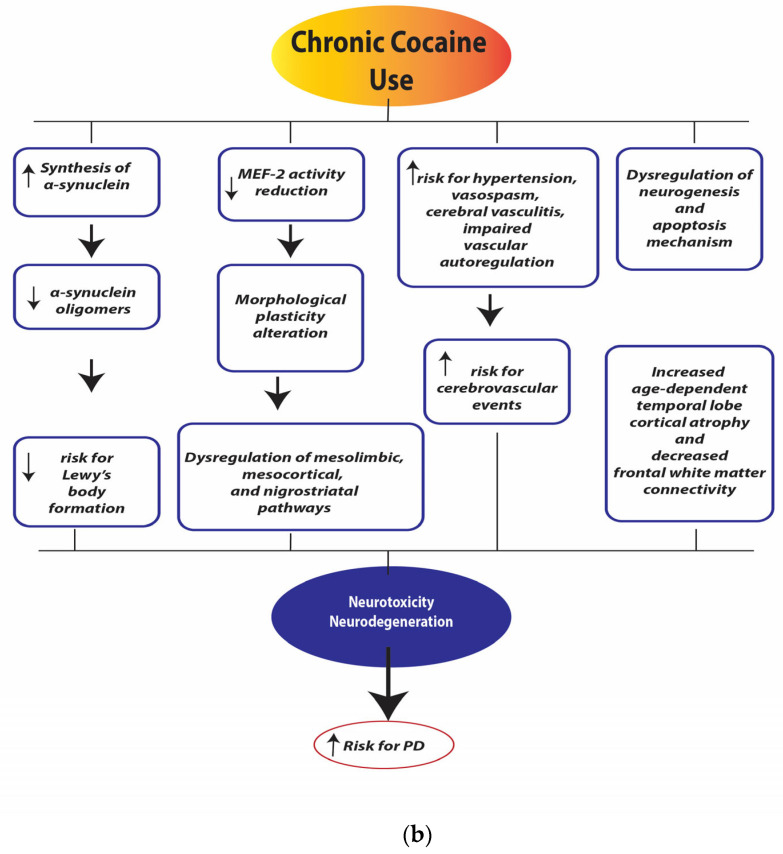
(**a**). Chronic cocaine use depletion of dopamine (DA), norepinephrine (NE), and serotonin (5-HT). Legend: dopamine transporter (DAT), norepinephrine transporter (NET), serotonin transporter (SERT), monoamine oxidase (MAO), reactive oxygen species (ROS), Vesicular Monoamine Transporter 2 (VMAT-2), dopamine receptor D_2_ (D_2_ receptors). (**b**). Chronic cocaine use neurotoxicity and neurodegeneration. Legend: myocyte enhancer factor-2 (MEF-2).

Figure 4a,b illustrate the neurobiological changes induced by chronic cocaine use.

The growing use of cocaine worldwide, affecting all social classes and various age groups, raises countless public health questions. The continuous use of the substance, the change in its quality, the methods of intake, and the association with other substances of abuse have detrimental effects not only on the CNS but on the entire organism. This results in the appearance of complex clinical pictures characterized by a varied symptomatological expression associated with morphological and structural organic alterations. It is the task of healthcare professionals in the clinical and research fields to know how to identify, recognize, and classify the signs and symptoms that cocaine users may present in the medium/long term. For example, knowing how to quickly recognize the potential neuropsychopathological and neuromotor side effects related to cocaine use could help to set up prevention, education, and treatment programs capable of reducing the potential destructive effects of the substance.

Starting from the assumption that we now have a lot of scientific evidence that correlates the continuous use of cocaine with an increased risk of the onset of neuropsychiatric manifestations, cognitive impairments, and neurodegenerative processes [158], in this article, we have tried to formulate an etiopathogenetic hypothesis of the effect of chronic cocaine use on the risk of developing PD.

As described above, the mechanisms responsible for cocaine neurotoxicity are complicated and may include excessive DA levels in the cytosol and synaptic cleft, mitochondrial dysfunctions, oxidative stress, neuroinflammation, pro-apoptotic changes, morphological plasticity alterations, and dysregulation of neurogenesis. Particularly, it is indicated that cocaine use causes damage to dopaminergic neurons and can develop symptoms of dopamine-related disorders and a 3-fold increased risk of motor symptoms [159,160,161].

Notably, alterations in α-synuclein expression, VMAT-2, and MEF-2 have mostly been identified as important mediators of neurotoxic mechanisms induced by cocaine [139,162]. Chronic cocaine intake directly increases α-synuclein levels, which in turn might indirectly promote tau phosphorylation processes. An inefficient and inadequate intracellular balance of α-synuclein would predispose individuals to the activation of and/or increase in neurodegeneration processes [100]. At the same time, the negative effects of chronic cocaine use on mitochondrial activity could be implicated in the genesis and/or maintenance of cytotoxicity and cell death processes; mitochondrial dysfunction is evident in many neurodegenerative processes, particularly in PD [80].

Functional and structural neuroimaging studies have indicated that the duration of cocaine use, accompanied by neurochemical, functional, and morphological changes, is strictly correlated with reduced grey matter density, specifically in the striatum, prefrontal cortex, and hippocampus [163,164,165,166].

This fact could explain the impairments in several cognitive functions, mostly attention, working memory, and executive function, which have been identified in long-term cocaine users [167,168,169,170]. Because antioxidant or anti-inflammatory agents have been shown to attenuate the risk for motor dysfunction and cognitive impairments, oxidative stress and neuroinflammatory changes might be implicated in cognitive and motor deficits and neurotoxicity induced by cocaine.

Even though the exact underlying pathogenesis mechanisms are not clear, it is possible that cocaine use may cause complex dopaminergic and mitochondrial dysfunction as well as increase the intracellular aggregation of α-synuclein and tau proteins, which, respectively, lead to synucleinopathies and tauopathies, and this, in turn, would activate a cascade of pro-inflammatory elements that would cause neuroinflammation and neurodegeneration. As highlighted by a recent study, the risk of developing neurodegenerative processes such as PD and Parkinsonism following chronic use of cocaine may also be related to specific genetic characteristics, suggesting a potential gene–environment interaction [171].

Finally, it is also worth considering another potential factor, namely the effect of chronic cocaine use on the gut microbiota (GM) and consequently on gut–brain communication. Microbial dysbiosis has recently been recognized as a factor related to substance use disorders (SUDs), and cognitive and neuropsychiatric disorders [172,173]. Cocaine, in addition to undermining the nutritional status of users, affects the GM and causes functional alterations in the immune system [174,175,176], gastrointestinal system [177,178,179,180,181,182], and, as described above, different neurotransmitter systems. In this sense, recently, it has been highlighted that cocaine addicts presented a compositional and functional dysbiosis of the microbiota (reduction in alpha diversity and modification of abundance of several taxa) and a dysregulation of many metabolic pathways, including the reduction in fecal butyric acid levels (a microbial-derived metabolite with anti-inflammatory effects) and an increase in medium-chain fatty acids with pro-inflammatory action [183]. The alterations of the GM and gut–brain axis induced by chronic cocaine use could therefore feed a peripheral and central pro-inflammatory state so as to favor the onset of neuropsychiatric and neurodegenerative disorders [184].

## 4. Conclusions

In conclusion, it could be possible to hypothesize that the chronic use of cocaine, in some subjects particularly at risk for neurodegenerative processes (due to genetic, environmental, cardio- and cerebrovascular factors, brain trauma, etc.), implying a condition of greater brain vulnerability, could induce the onset of a specific ***cocaine-related cerebropathy***. This condition would include not only motor symptoms but also cognitive symptoms attributable specifically to damage of dopaminergic pathways, such as a deficit in executive functions; this would lead to deficits in attentional control, cognitive inhibition, inhibitory control, working memory, cognitive flexibility, planning, and fluid intelligence (e.g., reasoning and problem solving). Furthermore, it would be very interesting to evaluate any potential correlations between neurodevelopmental disorders, chronic cocaine use, and the eventual onset of neurocognitive disorders. For example, we could try to evaluate whether subjects with ADHD, having a described greater predisposition to alterations in executive functions and to the development of the drug addiction process (often related to different reward mechanisms), could be more vulnerable to the neurotoxic effects of cocaine and therefore predisposed to the onset of the aforementioned cerebropathy. However, more studies are required to confirm this hypothesis.

Findings obtained from clinical and preclinical studies facilitated a better understanding of the cellular and molecular mechanisms involved in cocaine-induced neuropsychotoxicity and may provide targets for therapeutic intervention.

Although these conditions are not generally treatable, it is still important to correctly diagnose them as soon as possible and, at the same time, educate the population, medical health personnel, and users about the consequences of cocaine use.

## Data Availability

No new data were created in this study.
